# Antidepressants of the Serotonin-Antagonist Type Increase Body Fat and Decrease Lifespan of Adult *Caenorhabditis elegans*

**DOI:** 10.1371/journal.pone.0004062

**Published:** 2008-12-29

**Authors:** Kim Zarse, Michael Ristow

**Affiliations:** 1 Institute of Nutrition, University of Jena, Jena, Germany; 2 German Institute of Human Nutrition, Potsdam-Rehbrücke, Germany; L'université Pierre et Marie Curie, France

## Abstract

It was recently suggested that specific antidepressants of the serotonin-antagonist type, namely mianserin and methiothepin, may exert anti-aging properties and specifically extend lifespan of the nematode *C.elegans* by causing a state of perceived calorie restriction (Petrascheck M, Ye X, Buck LB: An antidepressant that extends lifespan in adult *Caenorhabditis elegans*; Nature, Nov 22, 2007;450(7169):553–6, PMID 18033297). Using the same model organism, we instead observe a reduction of life expectancy when employing the commonly used, standardized agar-based solid-phase assay while applying the same or lower concentrations of the same antidepressants. Consistent with a well-known side-effect of these compounds in humans, antidepressants not only reduced lifespan but also increased body fat accumulation in *C. elegans* reflecting the mammalian phenotype. Taken together and in conflict with previously published findings, we find that antidepressants of the serotonin-antagonist type not only promote obesity, but also decrease nematode lifespan.

## Introduction

In recent years, the nematorde *Caenorhabditis elegans* has become a well-established model organism to identify compounds that may be capable of extending lifespan not only in invertebrates, but also mammals. Accordingly, several research groups have published nematode-based findings on such compounds [1]–[30], whereas for most of these it is currently unknown whether they might exert similar effects in mammals, while for others this was proposed in regards to rodent lifespan [31] or at least in regards to reduction of aging-associated physiological alterations, whereas no extension of lifespan was observed [32].

Like numerous other psychoactive compounds, the antidepressant mianserin has been shown to increase appetite [33] as well as body mass [34] in humans. Conversely, obesity has been shown to decrease life span in humans [35] as well as *C. elegans* [25], while in both species serotonin signalling has been implicated in body fat accumulation [36]. In conflict with this evidence, recently published findings unexpectedly suggest that mianserin, and additional antidepressants of the serotonin antagonist type might extend *C.elegans* lifespan [24], which would surprisingly implicate that obesity promotes longevity.

While the latter study has employed liquid media to determine *C. elegans* lifespan, we have employed standardized and widely accepted agar-based assays aiming to replicate these findings, and unexpectedly observe a dose-dependent reduction of *C.elegans* lifespan, primarily suggesting that different assays to determine nematode lifespan generate opposing results.

## Results and Discussion

To replicate the findings of previously published experiments by Petrascheck and colleagues [24], we have we have applied both compounds described to be life-extending in the original paper, mianserin and methiothepin, to Bristol N2 *C.elegans* which in our case were maintained on solid-phase agar media, as described in Material and Methods.

**Figure 1:**
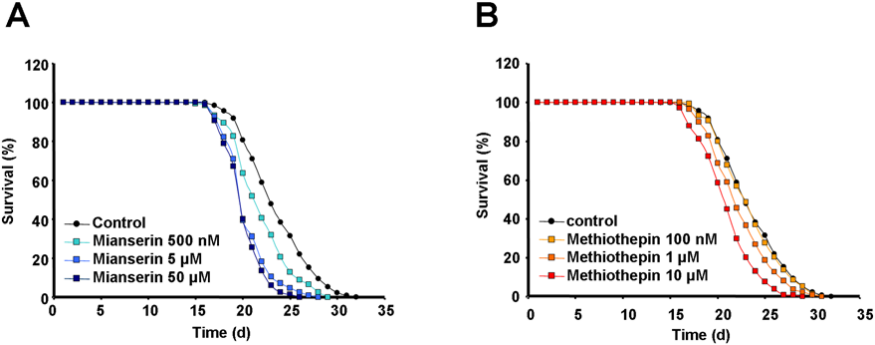
Antidepressants of the human serotonin antagonist type do not extend *Caenorhabditis elegans* lifespan. Panel A: The antidepressant mianserin shortens *C.elegans* lifespan at concentrations of 50 µM (dark blue boxes; this concentration was shown extend lifespan in the original publication [ref. 24]), 5 µM (medium blue boxes), and 500 nM (light blue boxes). Untreated control nematodes are depicted by black circles. Panel B: The chemically and functionally related compound methiothepin similarly shortens *C.elegans* lifespan at concentrations of 10 µM (red boxes; this concentration was shown extend lifespan in the original publication [ref. 24]), 1 µM (orange boxes), and has no significant effect on lifespan at a lower concentration of 100 nM (yellow boxes). Untreated control nematodes are depicted by black circles.

We repeatedly observed significantly *decreased* life expectancies for the key compound mianserin when applying this substance at a final concentration as given in the original paper (50 µM, p<0.001), as well as at 5 µM (p<0.001) and 500 nM (p<0.001) (Fig. 1a). Similar results were obtained for a functionally related compound, methiothepin, at concentrations of 10 µM (p<0.001) as well as at 1 µM (p<0.005), whereas this compound showed no significant effect at a concentration of 100 nM (Fig. 1b). Methiothepin was shown to extend life span in the original study at a concentration of 10 µM [24].

Petrascheck and colleagues have used liquid media not only for 96-well based screening assays, but also for final determinations of lifespans [24]. These liquid media are not commonly used for definite lifespan determinations, since they have been repeatedly reported to potentially cause differences in life span when compared to the well-established, standard solid-phase media; the first report in fact was published more the 30 years ago [37]. Liquid media have caused opposing results when being applied by different laboratories using apparently identical protocols [3], [9]. Moreover and according to their *Methods Summary* section [24], Petraschek *et al.* have not only based their liquid media on recipes from a publication [3] that was fundamentally put into question [9], but also from another laboratory [38] that has previously published a striking lack of correlation between lifespan results obtained with liquid- versus solid-phase media [39]. Lastly and most importantly, Petrascheck and colleagues observe a mean life expectancy of at least 23.6 days in N2 nematodes using their liquid media [24], whereas we [25] and others [40], [41] consistently observe a significantly shorter mean lifespan when using solid-phase media. This suggests that nematodes maintained in liquid media are kept in an *a priori* state of calorie restriction known to extend lifespan *per se*, *i.e.* in the absence of life-extending compounds [42], which has been recently shown to alter multiple pathways of energy metabolism [43] as to be expected in *a priori* states of calorie restriction [44]–[46].

**Figure 2:**
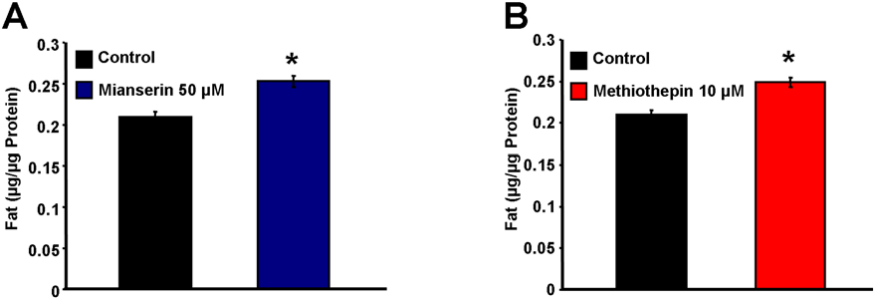
Antidepressants of the human serotonin antagonist type increase *Caenorhabditis elegans* body fat content. Panel A: The antidepressant mianserin increases *C.elegans* body fat content at a concentration of 50 µM (dark blue bar, right side; this concentration was shown extend lifespan in the original publication [ref. 24]) after ten days of treatment; untreated control nematodes are depicted as black bar. Panel B: The related compound methiothepin increases *C.elegans* body fat content at a concentration of 10 µM (red bar, right side; this concentration was shown extend lifespan in the original publication [ref. 24]) after ten days of treatment; untreated control nematodes are depicted as black bar.

Accordingly, and to test whether solid phase media as used in our *C.elegans* experiments reflect the situation in humans, we have tried to replicate the fact that mianserin increases human body mass [34] by applying this compound to nematodes. Indeed, both compounds significantly increased body fat after ten days of incubation at the concentrations that have been used by Petrascheck and colleagues [24] (Figs. 2a and 2b), whereas other pharmacological interventions known to extend *C.elegans* lifespan have been previously shown to decrease body fat content [25]. Nevertheless it should be noted that a specific genetic disruption that extends *C.elegans* lifespan, namely of the insulin-/IGF1-receptor signaling (*daf-2*) [40] have been shown to increase C.elegans body fat [47].

Taken together and consistent with the findings in humans in regards to obesity [33], [34], we find that antidepressants of the serotonin antagonist-type do not extend *C.elegans* lifespan at most commonly used and generally accepted experimental conditions.

## Materials and Methods

### Nematodes

The strain used in this study was Bristol N2 which was obtained from the Caenorhabditis Genetics Center (CGC, University of Minnesota, USA). Nematodes were grown and maintained on NGM agar plates as described previously [25], [48], [49]. All experiments were performed at 20° Celsius. *C. elegans* stocks and prefertile animals were maintained on OP50 bacteria.

### Compounds

Antidepressants mianserin and methiothepin were both obtained from Sigma-Aldrich (St. Louis, MO, USA). Agar plates containing experimental treatments were prepared from the same batch of NGM agar as the control plates except that the respective chemical was added to obtain the indicated final concentrations from a sterile stock solution (10 µM each).

### Fat content analyses

Triglyceride content was performed as previously described [25] briefly by flash-freezing nematodes and storage at −80°C until further processing. Approximately 25 mg was weighed and ground in a nitrogen-chilled mortar together with 250 µl of frozen phosphate buffer. The frozen material was gathered in a reaction tube and kept on ice. Extracts were sonicated three times and centrifuged for 7 min at 12,000 g. Fat content was determined with a commercially available triglyceride determination kit (Sigma-Aldrich) as previously described [50] and normalized to protein content, which was determined according to the Bradford method [51].
